# Oncolytic vaccinia virus reinvigorates peritoneal immunity and cooperates with immune checkpoint inhibitor to suppress peritoneal carcinomatosis in colon cancer

**DOI:** 10.1136/jitc-2020-000857

**Published:** 2020-11-16

**Authors:** Yu Seong Lee, Won Suk Lee, Chang Woo Kim, Seung Joon Lee, Hannah Yang, So Jung Kong, John Ning, Kyung-Mee Yang, Beodeul Kang, Woo Ram Kim, Hong Jae Chon, Chan Kim

**Affiliations:** 1 Department of Biomedical Science, CHA University, Seongnam, Korea (the Republic of); 2 Medical Oncology, CHA Bundang Medical Center, CHA University School of Medicine, Seongnam, Korea (the Republic of); 3 Kyung Hee University Gangdong Hospital, Gangdong-gu, Korea (the Republic of); 4 SillaJen Biotherapeutics, San Francisco, California, USA; 5 SillaJen, Busan, Korea (the Republic of)

**Keywords:** translational medical research, tumor microenvironment, immunotherapy, oncolytic virotherapy

## Abstract

**Background:**

Peritoneal carcinomatosis (PC) is a common and devastating manifestation of colon cancer and refractory to conventional anticancer therapeutics. During the peritoneal dissemination of colon cancer, peritoneal immunity is nullified by various mechanisms of immune evasion. Here, we employed the armed oncolytic vaccinia virus mJX-594 (JX) to rejuvenate the peritoneal antitumor immune responses in the treatment of PC.

**Methods:**

PC model of MC38 colon cancer was generated and intraperitoneally treated with JX and/or anti-programmed cell death protein 1 (PD-1) antibody. The peritoneal tumor burden, vascular leakage, and malignant ascites formation were then assessed. Tumors and peritoneal lavage cells were analyzed by flow cytometry, multiplex tissue imaging, and a NanoString assay.

**Results:**

JX treatment effectively suppressed peritoneal cancer progression and malignant ascites formation. It also restored the peritoneal anticancer immunity by activating peritoneal dendritic cells (DCs) and CD8^+^ T cells. Moreover, JX selectively infected and killed peritoneal colon cancer cells and promoted the intratumoral infiltration of DCs and CD8^+^ T cells into peritoneal tumor nodules. JX reinvigorates anticancer immunity by reprogramming immune-related transcriptional signatures within the tumor microenvironment. Notably, JX cooperates with immune checkpoint inhibitors (ICIs), anti-programmed death-1, anti-programmed death-ligand 1, and anti-lymphocyte-activation gene-3 to elicit a stronger anticancer immunity that eliminates peritoneal metastases and malignant ascites of colon cancer compared with JX or ICI alone.

**Conclusions:**

Intraperitoneal immunotherapy with JX restores peritoneal anticancer immunity and potentiates immune checkpoint blockade to suppress PC and malignant ascites in colon cancer.

## Background

The peritoneal cavity is an immunologically unique compartment.^1–5^ It has distinct immunological features, in comparison to systemic immunity, with relatively abundant dendritic cells (DCs), predominant CD8^+^ T cells over CD4^+^ T cells, and abundant soluble factors in peritoneal fluids, which establishes a robust immune competency.^4 6^ However, cancer cells can nullify this peritoneal immunity through various immune evasive mechanisms, and rapidly metastasize into the peritoneal cavity, making it the second most common site of metastasis in patients with colon cancer.^1 3 6–9^

During peritoneal dissemination of cancer cells, they are known to inactivate DCs and induce T cell exhaustion in the peritoneal cavity.^4 6 10^ Specifically, tumor cells overexpress PD-L1 on their surface and attenuate cell lysis induced by CD8^+^ cytotoxic T cells.^9^ Moreover, tumor cell-derived vascular endothelial growth factor (VEGF) generates malformed and leaky neovessels along the surface of the peritoneal cavity, hinders the effective delivery of anticancer drugs and cells, and promotes the accumulation of malignant ascites, in excess of immunosuppressive cytokines and immune cells.^4 11 12^ This unique tumor microenvironment (TME) of the peritoneal cavity severely disturbs the efficacy of conventional cytotoxic chemotherapeutics and targeted agents, leading to a poor prognosis in patients with peritoneal carcinomatosis (PC).^10 13 14^ Recently, to infiltrate this peritoneal TME, hyperthermic intraperitoneal chemotherapy was attempted in patients with colon cancer PC after complete cytoreductive surgery. However, the results did not show a meaningful survival benefit, and PC of colon cancer remains a significant clinical challenge.^15 16^

Over the past decade, cancer immunotherapy has emerged as a potent and effective therapeutic strategy for advanced cancers.^17–20^ Indeed, antibodies targeting immune checkpoints, including cytotoxic T-lymphocyte associated protein (CTLA)-4, programmed cell death protein 1 (PD-1), and programmed death-ligand 1 (PD-L1), elicit durable antitumor effects in patients with various malignancies.^21–24^ However, these immune checkpoint inhibitors (ICIs) showed limited efficacy as a monotherapy against colon cancer, especially peritoneal metastases.^19 23 25^ Therefore, a novel immunotherapeutic agent is urgently needed to overcome this limitation through the activation of peritoneal immunity.

Oncolytic virotherapy (OV) has been classified as a novel type of immunotherapy.^26–28^ It preferentially induces direct destruction of tumors through the selective infection and lysis of tumor cells.^27 29^ In this process, immunogenic cell death occurs with the widespread release of tumor-associated antigens, which are presented by DCs to activate antitumor immunity.^28^ Therefore, it acts as an in situ cancer vaccine within TME.^29^ In this regard, OV is an attractive therapeutic modality that can induce antigen-specific T cell response with the expansion of cytotoxic effector cells.^30^

Vaccinia virus, belonging to the poxvirus family, is highly immunogenic and has optimal characteristics that make it feasible for clinical development.^31 32^ JX-594 (pexastimogene devacirepvec, Pexa-vec) is an oncolytic vaccinia virus armed with GM-CSF, and it showed promising antitumor efficacies through its oncolytic, antiangiogenic, and immune-stimulating mechanisms, in both preclinical and clinical studies.^33–36^ Recently, we have reported that murine version of JX-594 (mJX-594, hereafter referred to as JX) robustly elicits the innate and adaptive immune responses within TME, thereby augmenting the efficacy of ICIs in immunotherapy-resistant kidney and breast cancers.^36^

Here, we demonstrate that intraperitoneal JX immunotherapy can rejuvenate peritoneal anticancer immunity, and enhance ICIs to suppress PC and malignant ascites in colon cancer.

## Methods

### Mice and cell lines

Male C57BL/6N mice between 8 and 12 weeks of age were purchased from Orient Bio Inc (Seongnam, Korea). Mice were housed in a specific pathogen-free animal facility at CHA University (Seongnam, Korea), and all experiments were approved by the Institutional Animal Care and Use Committee (IACUC, #180081) of CHA University.

The MC38 murine colon cancer cell line and ID8 ovarian cancer cell lines were obtained from the National Cancer Center (Goyang, Korea). MC38 and ID8 cells were cultured in Dulbecco’s modified Eagle’s medium (DMEM) supplemented with 10% fetal bovine serum (FBS) and 1% penicillin/streptomycin at 37°C, and was harvested at 80% confluence for the relevant experiments. The cell morphology and growth characteristics were conformant, and they were tested regularly for *Mycoplasma* using the MycoAlert Mycoplasma Detection Kit (Lonza, New Jersey, USA).

### Construction and production of virus

JX, provided by SillaJen Inc (Seoul, Korea), is a Western Reserve strain of the vaccinia virus encoding murine GM-CSF in the vaccinia thymidine kinase gene locus under the control of the p7.5 promoter.^37 38^ The generation and quantification of the virus were previously described.^36^ The virus titer was determined using a plaque assay of U-2 OS cells.

### PC model and treatment regimens

To generate peritoneal tumors, we intraperitoneally injected either 5 × 10^5^ MC38 colon cancer cells or 1.5 × 10^7^ ID8 ovarian cancer cells into the peritoneal cavity of wild-type C57BL/6 mice. Tumor-implanted mice were randomized to each experimental group 7 days after implantation. Mice were treated with an intraperitoneal injection of 1 × 10^7^ plaque-forming units (pfu) of JX. For combination immunotherapy, we also administered anti-PD-1 (10 mg/kg, clone J43, BioXCell), anti-VEGFR2 (25 mg/kg, clone DC101, BioXCell), anti-PD-L1 (10 mg/kg, clone 10F.9G2, BioXCell), and anti-LAG-3 (10 mg/kg, clone C9B7W, BioXCell) intraperitoneally at given time points. The optimal doses for checkpoint blockade were determined from previous studies.^36 39^ Mice in the control group were treated with an intraperitoneal injection of the same volume of phosphate-buffered saline (PBS). Tumor-bearing mice were weighed twice weekly and monitored daily for the clinical sign of swollen bellies indicative of ascites formation. During the sacrifice, ascitic fluid was aspirated entirely directly from the peritoneal cavity of all mice using a 26-gauge needle. The tumor nodules in the peritoneal cavity and peritoneum were harvested and weighed, and peritoneal cells were prepared performing a peritoneal lavage by washing the peritoneum with 3 mL of 3% FBS in PBS, containing 2 mmol/L EDTA. The survival of each mouse was monitored, and the overall survival was calculated.

### Flow cytometry analysis of tumor-associated immune cells

For flow cytometry analysis, harvested tumors were minced into small pieces with scissors and incubated in digestion buffer, comprised of 2 mg/mL collagenase D (COLLD-RO, Roche) and 40 µg/mL DNase I (10104159001, Roche), for 1 hour at 37°C. The cell suspensions were filtered through a 70 µm cell strainer (352350, Falcon) and incubated for 3 min at room temperature in ammonium chloride-potassium lysis buffer (A1049201, Gibco) to remove cell clumps and red blood cells. After washing with PBS, the cells were filtered through a 40 µm nylon mesh and resuspended in FACS buffer (1% FBS in PBS). Peritoneal cells, collected from the peritoneal cavity using lavage, were lysed with ACK buffer as described above. In the same way, the cells were filtered and resuspended in FACS buffer. Next, single-cell suspension isolated from tumor tissues and peritoneal cavity were incubated on ice for 30 min in Fixable Viability Dye eFluor^TM^ 450 (65-0863-18, eBioscience) to exclude dead cells before antibody staining. Then the cells were washed with FACS buffer and incubated with mouse Fc receptor binding inhibitor (CD16/32, clone 2.4G2, BD Pharmingen) for 15 min at room temperature before staining with surface antibodies against CD45 (clone 30-F11, BD Pharmingen), CD3 (clone 17A2, eBioscience), CD4 (clone RM4-5, eBioscience) and CD8 (clone 53-6.7, eBioscience) for 30 min on ice. Cells were further permeabilized using a FoxP3 fixation and permeabilization kit (eBioscience), and stained for FoxP3 (clone FJK-16s, eBioscience) or Granzyme B (clone NGZB, eBioscience). For intracellular cytokine staining, cells from peritoneal cavity were stimulated for 4 hours with 20 ng/mL PMA (Sigma) and 1 μM Ionomycin (Sigma) in the presence of 3 µg/mL Brefeldin A (eBioscience). After stimulation, cells were fixed, permeabilized, and stained for interferon (IFN)-γ (clone XMG1.2, eBioscience) and TNF-α (clone MP6-XT22, BD Pharmingen). Tumor cells (CD45^−^CD31^−^), CD4^+^ T cell (CD45^+^CD4^+^), CD8^+^ T cell (CD45^+^CD8^+^), DCs (CD45^+^CD11c^+^), myeloid cell (CD45^+^CD11b^+^) and Tregs (CD4^+^CD25^+^) were sorted from tumors using MoFlo XDP cell sorter (Beckman Coulter). Flow cytometry was performed using a CytoFLEX flow cytometer (Beckman Coulter) and analyzed using FlowJo software (Tree Star Inc, Ashland, Oregon, USA).

### Immune-related gene expression profiling using NanoString

Total RNA was extracted from fresh tumor tissues using TRIzol reagent (Invitrogen) and purified with ethanol. RNA concentration and quality were confirmed with a Fragment Analyzer instrument (Advanced Analytical Technologies, Iowa, USA). We used 100 ng of total RNA from each tumor sample for the digital multiplexed profiling with NanoString nCounter PanCancer Immune Profiling mouse panel (NanoString Technologies), as per our previously established protocol.^36^

### Gene expression analysis using RT^2^ Profiler PCR array

Total RNA was extracted from sorted cells using the TRIzol reagent (Invitrogen) and purified with ethanol. RNA was reverse-transcribed using the GoScript Reverse Transcription kit (Promega) for cDNA synthesis and then loaded on to RT^2^ Profiler PCR Array Mouse Cancer Inflammation and Immunity Crosstalk according to manufacturer’s instructions (Qiagen). Reactions were performed in the LightCycler96 (Roche) for 10 min at 95°C followed by 45 cycles of 15 s at 95°C and 1 min at 60°C.

### Enzyme-linked immunosorbent assay (ELISA)

To quantify damage-associated molecular patterns (DAMPs), 4 × 10^5^ MC38 cells seeded into six-well plates overnight were infected with JX at MOIs of 0 and 10 in 10% FBS-containing DMEM for 24 hours. Culture supernatants were harvested to measure calreticulin and HMGB1, and cell pellets were lysed to measure annexin A1. Calreticulin (Biomatik, Ontario, California, USA), HMGB1 (Biomatik, Ontario, CA), and annexin A1 (Abcam, Burlingame, California, USA) were quantified using the ELISA kit following the manufacturer’s instructions.

### IFN-γ enzyme-linked immunospot (ELISPOT) assay

For the quantification of tumor-specific cytotoxic T cells, splenocytes, tumor-infiltrating lymphocytes (TILs), and peritoneal cells from each group were isolated 5 days after the last treatment. Harvested cells were labeled using the Dead Cell Removal Kit (Miltenyi Biotec, Auburn, 130090101) according to the manufacturer’s instructions, and purified using MACS (Miltenyi Biotec, Auburn, California, USA). Purified live cells were stimulated with MC38 tumor cells at a 10:1 ratio in mouse IFN-γ-precoated 96-well plates (MABTECH AB, Nacka Strand, Sweden), which were then incubated for 36 hours at 37°C in a CO_2_ incubator. After being washed, plates were stained with 1 µg/mL of the biotinylated antimouse IFN-γ antibody, R4-6A2-biotin, for 2 hours, followed by incubation with a streptavidin-ALP solution for 1 hour at room temperature. After the addition of BCIP/NBT-plus substrate solution, the number of spots was counted using ImageJ software (http://rsb.info.nih.gov/ij).

### Histologic analysis via immunofluorescence

For immunofluorescence staining, tumor samples were fixed in 1% PFA at room temperature and were washed several times with PBS, dehydrated overnight with 20% sucrose, and embedded in tissue-freezing medium (Leica). Frozen tissues were sectioned into 50 µm thick blocks, which were permeabilized with 0.3% PBS-T (Triton X-100 in PBS), and then blocked with 5% goat serum in 0.1% PBS-T for 1 hour. Next, the samples were incubated overnight with the following primary antibodies: Rabbit anti-vaccinia virus (Abcam), hamster (clone 2H8, Millipore) and rabbit anti-CD31 (Abcam), rat anti-CD8 (clone 53–6.7, BD Pharmingen), rat anti-CD4 (clone RM4-5, Invitrogen), hamster anti-CD11c (clone HL3, BD Pharmingen), rat anti-Granzyme B (clone NGZB, eBioscience), rat anti-IFN-γ (clone XMG1.2, eBioscience), rat anti-TNF-α (clone MP6-XT22, BD Pharmingen) or rat anti-FoxP3 (clone FJK-16s, eBioscience). After several washes, the samples were incubated for 2 hours at room temperature with the following secondary antibodies: FITC-conjugated, Cy3-conjugated, or Cy5-conjugated anti-rabbit IgG (Jackson ImmunoResearch), FITC-conjugated anti-rat IgG (Jackson ImmunoResearch), FITC-conjugated or Cy3-conjugated anti-hamster IgG (Jackson ImmunoResearch). The cell nuclei were counterstained with 4′6-diamidino-2-phenylindole (Invitrogen). Finally, samples were mounted with fluorescent mounting medium (DAKO), and images were acquired using an LSM 880 confocal microscope (Carl Zeiss).

### Morphometric analysis

Density measurements of the JX, blood vessels, T lymphocytes, and DCs in the same area were performed using ImageJ software. The JX^+^ density per random 0.49 mm^2^ area was calculated in tumor sections to determine the level of JX infection. Blood vessel density was measured by calculating the CD31^+^ density per random 0.49 mm^2^ area in tumor sections. The degree of cytotoxic T lymphocyte infiltration was calculated as the percentage CD8^+^ and CD4^+^ per random 0.49 mm^2^ area in intratumoral regions. The density of DCs was determined by calculating the percentage CD11c^+^ in random 0.49 mm^2^ area. All measurements were performed in at least five areas per mouse.

### Statistical analysis

Statistical analyses were performed using GraphPad Prism V.7.0 software (GraphPad Software, La Jolla, California, USA) and PASW statistics V.18 (SPSS). Values are represented as mean±SE unless otherwise indicated. The Shapiro-Wilk normality test was performed for all datasets to analyze whether each dataset follows a normal distribution pattern. If the dataset followed a normal distribution, we applied parametric tests such as the Student’s t-test and one-way analysis of variance. If the dataset did not follow normal distribution owing to small sample size, we used non-parametric tests such as the Mann-Whitney U test and Kruskal-Wallis test. Adjusted p values were used to analyze Nanostring data according to the Benjamini-Hochberg method. Gene set enrichment analysis was used to test the enrichment of a specific gene set, and core enrichment genes were determined. Survival curves were plotted using the Kaplan-Meier method, and statistical differences between curves were analyzed using the log-rank test. The level of statistical significance was set at p<0.05.

## Results

### JX treatment restores the peritoneal immunity

**Figure 1:**
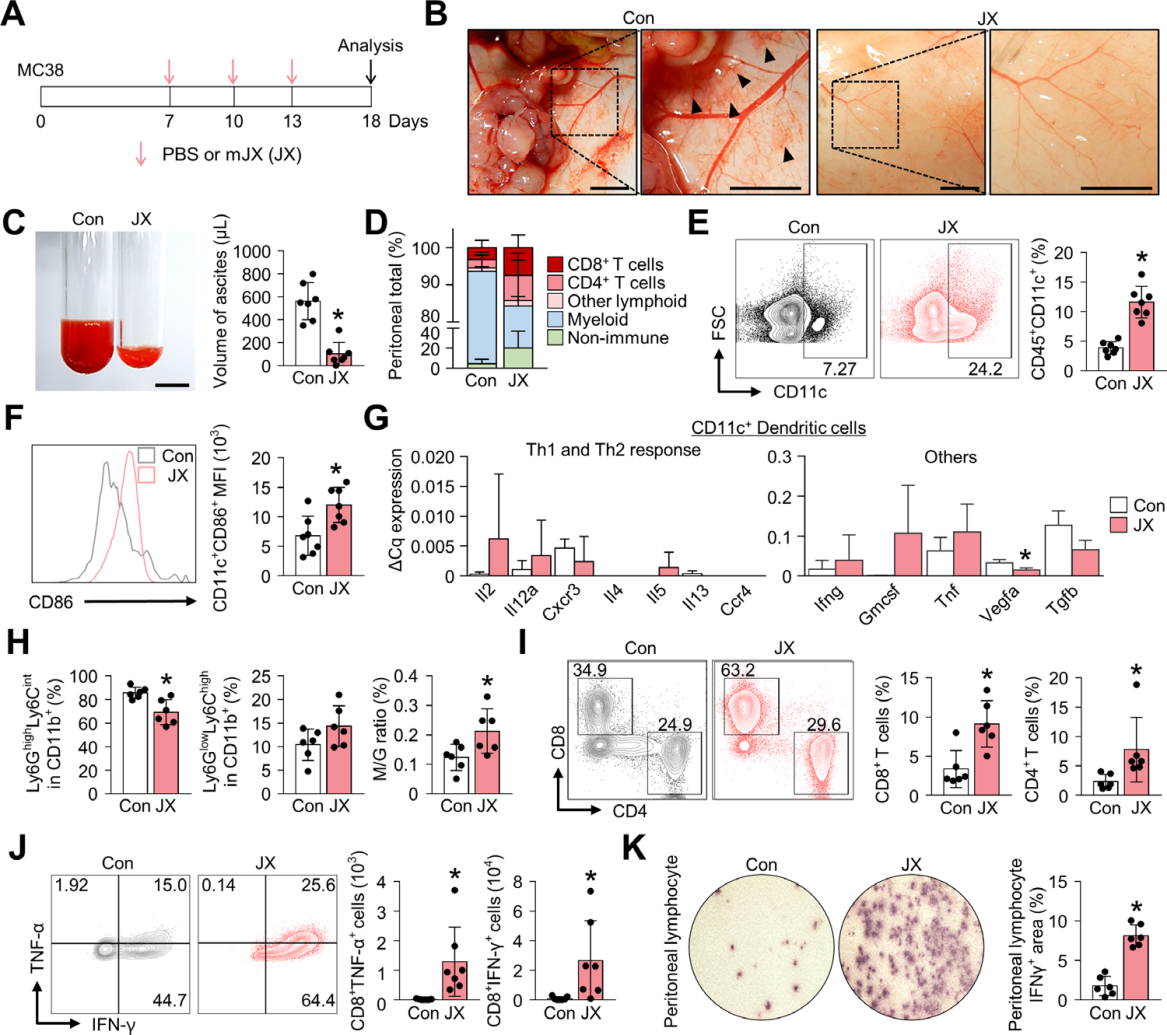
JX treatment activates the peritoneal immunity. MC38 colon tumor cells were implanted intraperitoneally into mice, which were treated intraperitoneally with PBS or mJX-594 (JX, 1 × 10^7^ pfu) three times. (A) Diagram of the treatment schedule. Red arrows indicate treatment with intratumoral delivery of JX, and black arrows indicate sacrifice. (B) Representative images of the parietal peritoneum and its blood vessels. Black arrowheads indicate peritoneal hemorrhages. (C) Representative images and comparisons of malignant ascites in PBS-treated or JX-treated mice. (D) Diagram depicting the comparison of the peritoneal cell population. (E and F) Representative flow cytometric plot and comparisons of CD11c^+^ DCs (E) and their CD86 expression (F). (G) Comparisons of cytokine and chemokine expressions in CD11c^+^ DCs in PBS-treated and JX-treated tumors. Intratumoral DCs were isolated using fluorescence-activated cell-sorting. (H) Comparisons of fractions of Ly6G^high^Ly6C^int^ granulocytic myeloid cells and Ly6G^low^Ly6C^high^ monocytic myeloid cells. (I) Representative flow cytometric plot and comparisons of CD8^+^ and CD4^+^ T cell fractions. (J) Representative flow cytometric plot and comparisons of TNF-α and IFN-γ-expressing fraction in CD8^+^ T cells. (K) Images and comparisons of IFN-γ ELISPOT in peritoneal CD8^+^ T lymphocytes from PBS or JX-treated mice. Pooled data from two experiments with n=6 to 7 per group. Values are mean±SD. *p<0.05 versus control. Two-tailed Student’s t*-*test and Mann-Whitney U test were used (C and E–K). Scale bars, 5 mm (B), 10 mm (C). DCs, dendritic cells; PBS, phosphate-buffered saline; pfu, plaque-forming units.

The PC model was established via the intraperitoneal injection of MC38 colon cancer cells into C57BL/6 mice. One week after tumor implantation, mice were treated with intraperitoneal injections of either JX or PBS (figure 1A). At day 18 of tumor implantation, all PBS-treated control mice developed multiple peritoneal seeding metastases on the surface of the visceral and parietal peritoneum and accumulated malignant ascites in the peritoneal cavity (figure 1B). In PBS-treated mice, malformed neovessels were observed on the peritoneal surface that is close to the tumor mass, and these vessels were accompanied by the foci of small peritoneal hemorrhages. In contrast, JX-treated mice had a remarkably fewer number of tumor nodules which were smaller compared with that of the control mice in the peritoneal cavity, and the volume of malignant ascites was 82% less compared with that of the control mice (figure 1B, C). Moreover, there were fewer neovessels on the peritoneal surface, and less peritoneal hemorrhage was observed, indicating suppressed angiogenesis by JX in the peritoneal cavity (figure 1B). Intraperitoneal JX treatment was generally well-tolerated, except for the transient and slight weight loss (~1 g) within 3 days of the first injection, which was self-limited and completely resolved within a week. There were no treatment-related mortalities.

In parallel, flow cytometry analyses revealed the alteration of innate and adaptive immunity in the peritoneal cavity (figure 1D). At first, JX treatment increased the number of intraperitoneal CD11c^+^ DCs by 3.0-fold (figure 1E). Notably, the expression of CD86 was enhanced in CD11c^+^ DCs, suggesting the activation of peritoneal DCs after JX treatment (figure 1F). Intriguingly, DCs within JX-treated tumors induced Th1-predominant responses compared with those within PBS-treated tumors. Moreover, JX-activated DCs upregulated *Gmcsf*, *Tnf*, *Ifng*, while downregulating *Vegfa* and *Tgfb* (figure 1G). In addition, the analysis of CD11b^+^ myeloid cells showed a significant decrease in the number of Ly6G^high^Ly6C^int^ granulocytic cells with a slight increase in the number of Ly6G^low^Ly6C^high^ monocytic cells, resulting in an increased ratio of the monocytic myeloid cells to granulocytic myeloid cells (figure 1H).

Finally, regarding the adaptive immunity in the peritoneal cavity, JX treatment increased the number of peritoneal CD8^+^ cytotoxic T cells by 2.7-fold and that of CD4^+^ T cells by 3.3-fold compared with the control group (figure 1I). Indeed, the number of TNF-α- or IFN-γ-secreting CD8^+^ T cells were also dramatically increased after the JX treatment (figure 1J). IFN-γ ELISPOT assay was also performed using peritoneal lymphocytes in the presence of MC38 tumor cells to confirm the tumor-specific effector function of the intraperitoneal T cells after JX treatment, and IFN-γ spots were observed 4.62-fold more frequently in the JX-treated mice compared with the control (figure 1K). Collectively, these findings indicate that the JX treatment effectively suppressed the peritoneal progression of colon cancer and malignant ascites formation, via enhanced innate and adaptive immunity in the peritoneal cavity.

### JX treatment suppresses tumor angiogenesis and facilitates immune cell infiltration into the tumor

**Figure 2:**
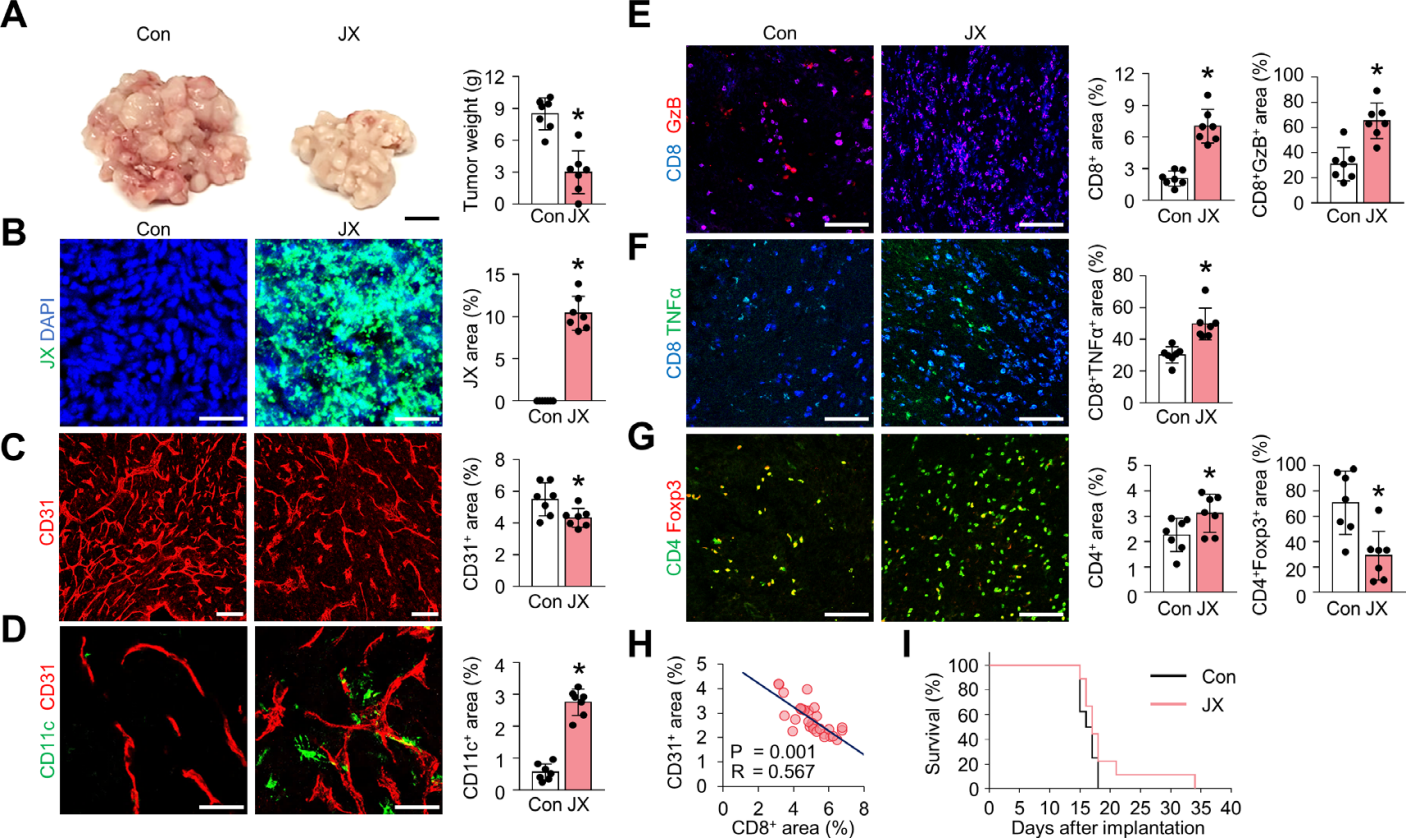
JX treatment reduces peritoneal metastases by suppressing angiogenesis and promoting immune cell infiltration into peritoneal tumors. MC38 tumor-bearing mice were intraperitoneally treated with PBS or JX. (A) Representative images and comparisons of tumor burden in the PBS-treated or JX-treated mice. (B–D) Representative images and comparisons of JX infection (B), CD31^+^ tumor blood vessels (C), and CD11c^+^ DCs (D). (E–G) Representative images and comparisons of CD8^+^GzB^+^ T cells (E), CD8^+^TNF-α^+^ T cells (F), and CD4^+^Foxp3^+^ T cells (G) within tumors. (H) Correlation between intratumoral CD8^+^ T cells and CD31^+^ blood vessels in JX-treated tumors (n=30). (I) Kaplan-Meier survival curves for overall survival in PBS-treated or JX-treated mice. Unless otherwise denoted, pooled data from two experiments with n=7 to 8 per group. Values are mean±SD. p<0.05 versus control. Two-tailed Student’s t test and Mann-Whitney U test were used (A–G). Scale bars, 10 mm (A), 50 µm (B and D), 100 µm (C and E–G). DCs, dendritic cells; GzB, granzyme B; PBS, phosphate-buffered saline.

To confirm the effect of JX on TME, we analyzed tumor-infiltrating immune cells within the peritoneal tumors. After consecutive injections of JX, intraperitoneal tumor burden was reduced by 65% when compared with the control group (figure 2A). Consistent with previous reports,^36 37 40^ JX selectively infected and destroyed colon cancer cells but also disrupted tumor blood vessels within TME (figure 2B, C). These JX-infected, dying tumor cells released DAMPs, such as calreticulin and annexin A1, which are involved in the activation of DCs (online supplemental figure S1).^41 42^ Additionally, JX itself further induced the activation and maturation of DCs within TME because it was genetically engineered to secrete mGM-CSF. Accordingly, the number of intratumoral DCs were markedly increased by 3.9-fold compared with that of the PBS-treated tumors (figure 2D). Furthermore, the intratumoral infiltration of CD8^+^ T cells increased 3.4-fold, and most cells upregulated the expression of granzyme B (GzB) and TNF-α after JX treatment (figure 2E, F). FoxP3-expressing CD4^+^ T cells decreased by 59% with intraperitoneal JX treatment (figure 2G). The JX-induced anti-angiogenic effect also correlated with increased CD8^+^ T cells within peritoneal tumors (figure 2H). The degree of aberrant tumor vasculatures suppressed with JX treatment correlated with the number of CD8^+^ cytotoxic T cells infiltrated into TME. Although JX monotherapy-induced dramatic remodeling of TME, it did not show a remarkable survival benefit as a monotherapy, suggesting the need for optimal combination partners for JX (figure 2I).

10.1136/jitc-2020-000857.supp1 Supplementary data

Overall, these results suggested that JX could suppress tumor angiogenesis and promote the infiltration of CD11c^+^ DCs and CD8^+^ T cells into tumor nodules, eliciting an effective antitumor immune response in TME.

### JX treatment reinvigorate effector functions of intratumoral T cells

**Figure 3:**
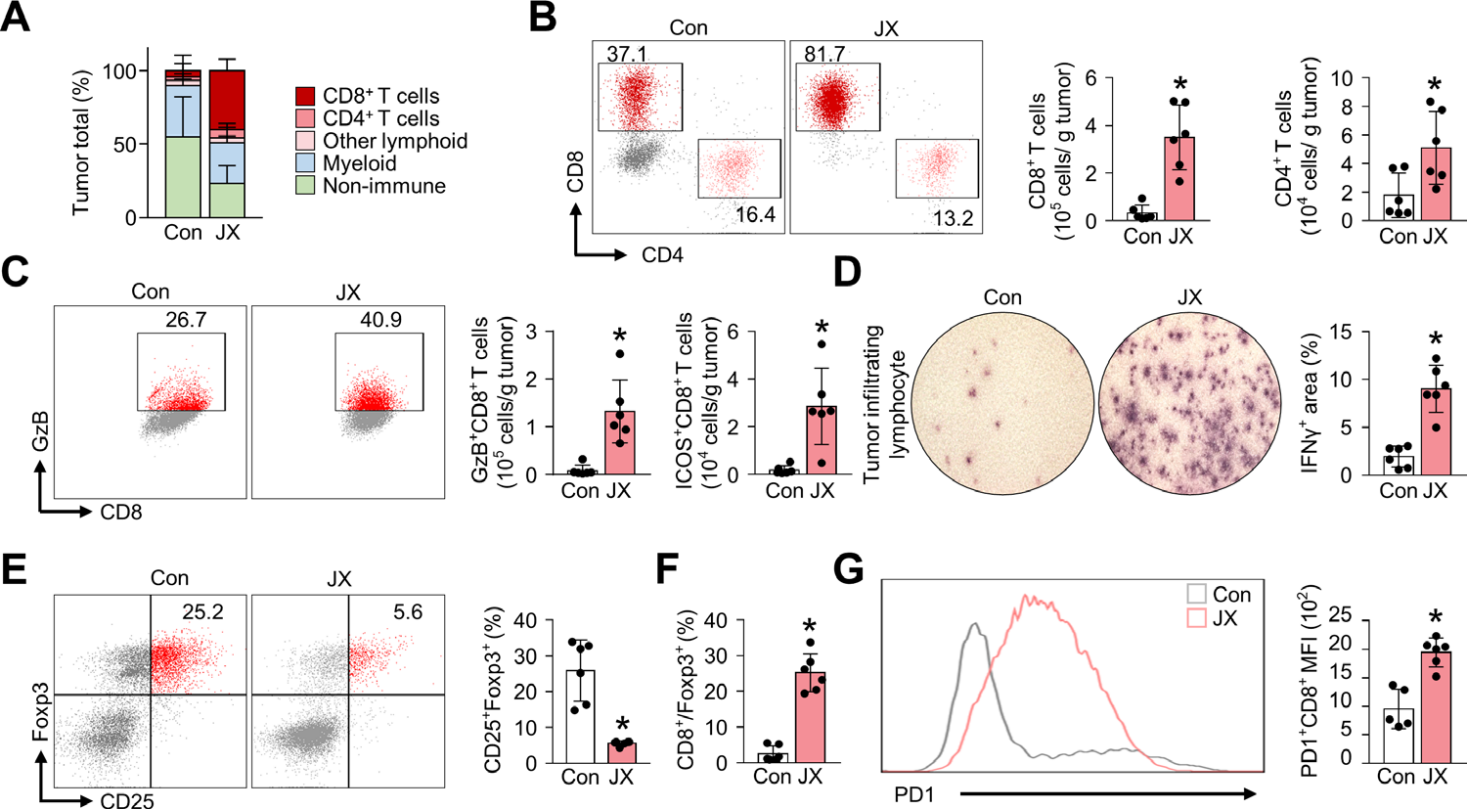
JX reinvigorate the effector function of tumor-infiltrating T cells. Mice were implanted with MC38 tumor cells and treated with intraperitoneal injections of PBS or JX. (A) Diagram depicting the comparison of the tumor-infiltrating cell population. (B) Representative flow cytometric images and comparisons of CD8^+^ and CD4^+^ T cell fractions. (C) Comparisons of fractions of CD8^+^GzB^+^ and CD8^+^ICOS^+^ cells. (D) Images and comparisons of IFN-γ ELISPOT in CD8^+^ tumor-infiltrating lymphocytes from PBS-treated or JX-treated mice. (E) Representative flow cytometric plot showing CD25^+^Foxp3^+^ (Treg) fraction in CD4^+^ T cells. (F) Comparisons of CD8/Treg ratio in the tumor. (G) Representative histogram showing PD-1 expression in CD8^+^ T cells. Pooled data from two experiments with n=5 to 7 per group. Values are mean±SD. p<0.05 versus control. Two-tailed Student’s t-test and Mann-Whitney U test were used (B–G). ELISPOT, enzyme-linked immunospot; GzB, granzyme B; PBS, phosphate-buffered saline; PD-1, programmed cell death protein 1.

In order to evaluate the role of JX on TME further, we compared the phenotype of tumor-infiltrating T cells in control or JX-treated tumors. First, JX treatment notably increased the number of intratumoral lymphocytes by 6.6-fold compared with control mice, while the number of myeloid cells was decreased by 22% (figure 3A). In particular, the number of CD8^+^ and CD4^+^ T cells were increased by 10.6-fold and 2.9-fold, respectively, compared with that of the control group (figure 3B). To evaluate the activation status of intratumoral CD8^+^ T cells, we assessed the expression of GzB and inducible T-cell costimulator (ICOS), which are known as the T cell activation and costimulatory markers.^36^ In CD8^+^ T cells of JX-treated tumors, the expression of GzB was increased 35.3-fold, and the expression of ICOS was also upregulated by 16.0-fold compared with that of control tumors (figure 3C).

Additionally, the TILs in JX-treated tumors secreted increased levels of IFN-γ (4.7-fold) compared with the control tumors (figure 3D). Also, the number of CD4^+^CD25^+^Foxp3^+^ regulatory T cells (Treg) was reduced by 78.3% in JX-treated tumors compared with that of the control tumors (figure 3E). Moreover, the ratio of cytotoxic T cells to Tregs was increased by 9.7-fold after JX treatment, indicating the increase in the overall effector function of the intratumoral T cells (figure 3F). On the other hand, although JX activated anticancer immunity, it also significantly increased the number of inhibitory checkpoint molecules, such as PD-1 in CD8^+^ T cells, suggesting the induction of negative feedback mechanism to counter-balance immune response in the TME (figure 3G). Collectively, these findings demonstrated that the JX treatment rejuvenated the effector functions of CD8^+^ T cells in TME, thereby generating a robust antitumor immunity.

### JX reprograms the immune-related transcriptional signature in peritoneal metastasis

**Figure 4:**
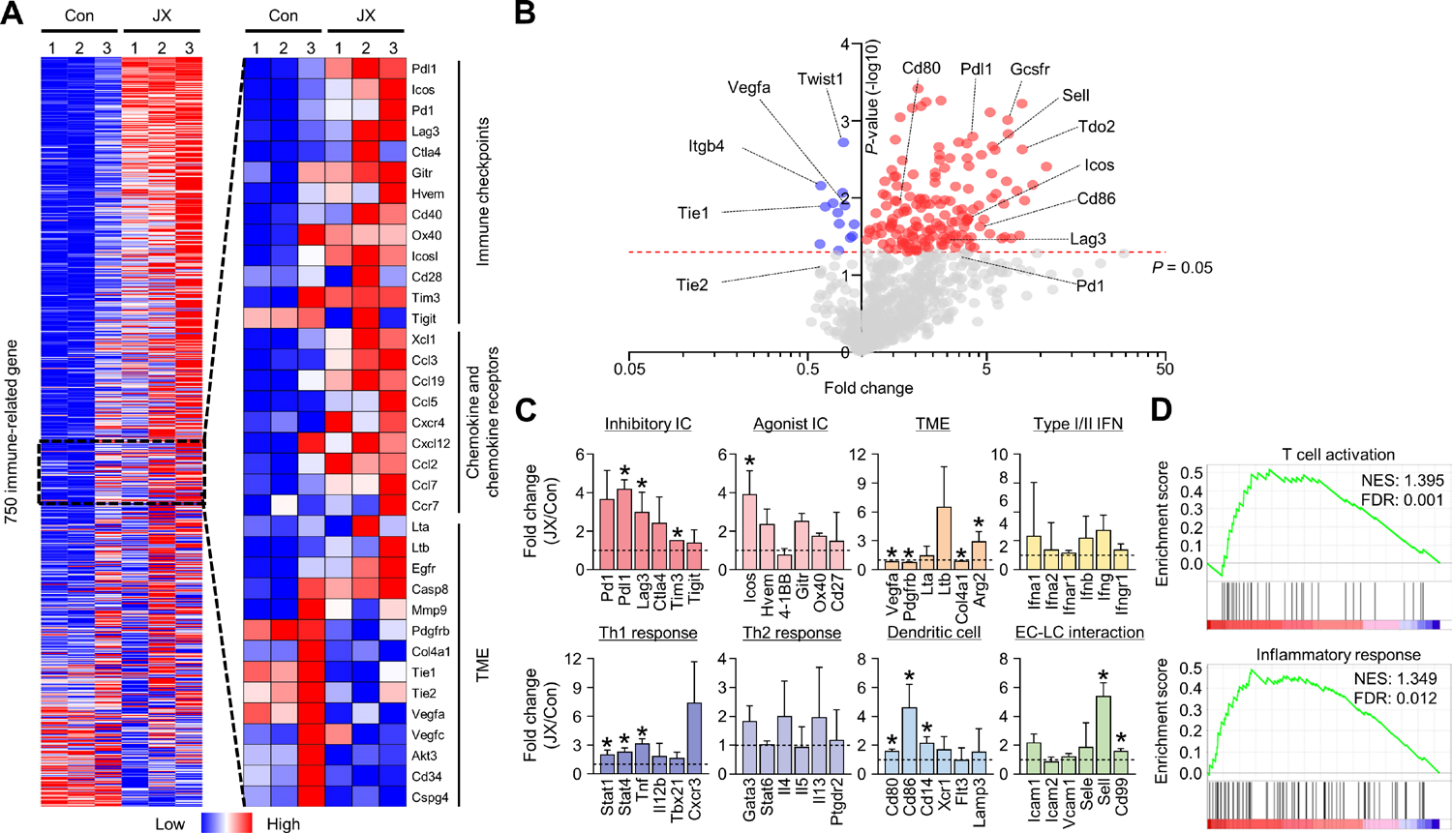
JX reprograms the peritoneal TME to T cell-inflamed tumors. MC38 tumor cells were implanted intraperitoneally into mice and treated with intraperitoneal injections of PBS or JX. (A) NanoString immune-related gene expression heat map. Red and blue color represent upregulated and downregulated genes, respectively. (B) Volcano plot showing the change of gene expression profile in JX-treated tumors of mice. Red line indicates p<0.05. (C) Comparisons of gene expressions related to inhibitory and agonist immune checkpoints, TME, type I/II IFNs, Th1 response, Th2 response, DC, and endothelial cell-lymphocyte interaction. (D) GSEA of gene sets involved in T cell activation and an inflammatory response. Pooled data from two experiments with n=3 per group. Values are mean±SD. p<0.05 versus control. Two-tailed Student’s t-test was used (C). DC, dendritic cell; GSEA, gene set enrichment analysis; PBS, phosphate-buffered saline; TME, tumor microenvironment.

To comprehensively analyze the JX-induced immune changes in the peritoneal metastases of colon cancer, we assessed the transcriptional changes of immune-related genes using the NanoString PanCancer Immune profiling panel. The results revealed distinct immunologic reprograming in response to JX treatment, especially in immune checkpoint molecules, chemokines, and chemokine receptors (figure 4A, online supplemental table 1). Overall, 102 genes were upregulated more than twofold after JX monotherapy (figure 4B). Notably, the genes related to immune checkpoint (*Pdl1, Lag3, Tim3*), TME (*Arg2*), Th1 (*Stat1, Stat4, Tnf*), DCs (*Cd80, Cd86, Cd14*), and endothelial-lymphocyte interaction (*Sell, Cd99*) were significantly upregulated in JX-treated tumors compared with the control tumors (figure 4C). Intriguingly, intratumoral *Vegfa* expression was significantly suppressed after JX treatment, which is consistent with our observation of reduced neovessels in the peritoneal cavity. Furthermore, gene sets related T cell activation and inflammatory response were more enriched in JX-treated tumors compared with control tumors (figure 4D). JX treatment induced differential changes in various cell populations. JX upregulated *Ifng*, *Tnf*, and *Gmcsf* in most of immune cells, but not in tumor cells. Myeloid cells and DCs upregulated Th1 cytokines. *Pdl1* expression was increased in most of immune cells, whereas *Pd1* upregulation was observed in CD8^+^ and CD4^+^ T cells (online supplemental figure S2).

10.1136/jitc-2020-000857.supp2 Supplementary data

Consequentially, these results demonstrated that immune checkpoints, Th1 response, and type I/II IFN-related genes in the intraperitoneal seeding nodules were extensively reprogrammed in response to JX treatment, thereby converting non-inflamed peritoneal tumors to T cell-inflamed tumors.

### JX cooperates with PD-1 blockade to elicit potent anticancer immunity that eliminates peritoneal metastases of colon cancer

**Figure 5:**
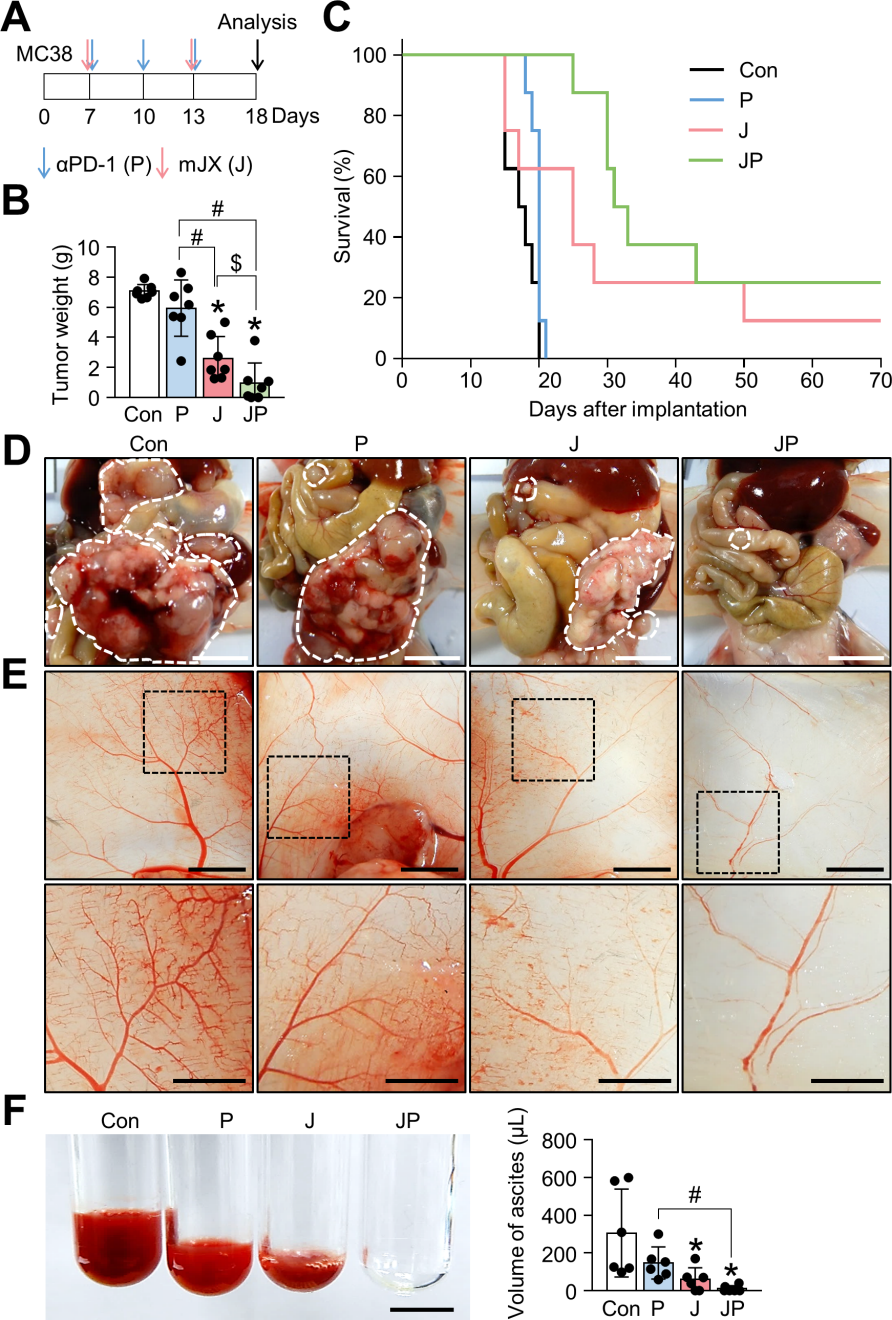
JX cooperates with PD-1 blockade to suppress PC and malignant ascites formation in colon cancer. Mice were intraperitoneally implanted with MC38 tumor cells and treated with JX and/or anti-PD-1 on indicated days (arrows). Black arrow indicates mice sacrifice. (A) Diagram of the treatment schedule. (B) Comparisons of tumor weight in mice. (C) Kaplan-Meier survival curves for overall survival. (D) Representative images and comparisons of peritoneal tumors. (E) Representative images and comparisons of the parietal peritoneum and its blood vessels. (F) Representative images and comparisons of malignant ascites. Pooled data from two experiments with n=6 to 8 per group. Values are mean±SD *p<0.05 versus control; ^#^p<0.05 versus anti-PD-1; ^$^p<0.05 versus JX. Kruskal-Wallis test was used (B and F). Scale bar, 5 mm (D and E) and 2.5 mm (F). PC, peritoneal carcinomatosis; PD-1, programmed cell death protein 1.

**Figure 6:**
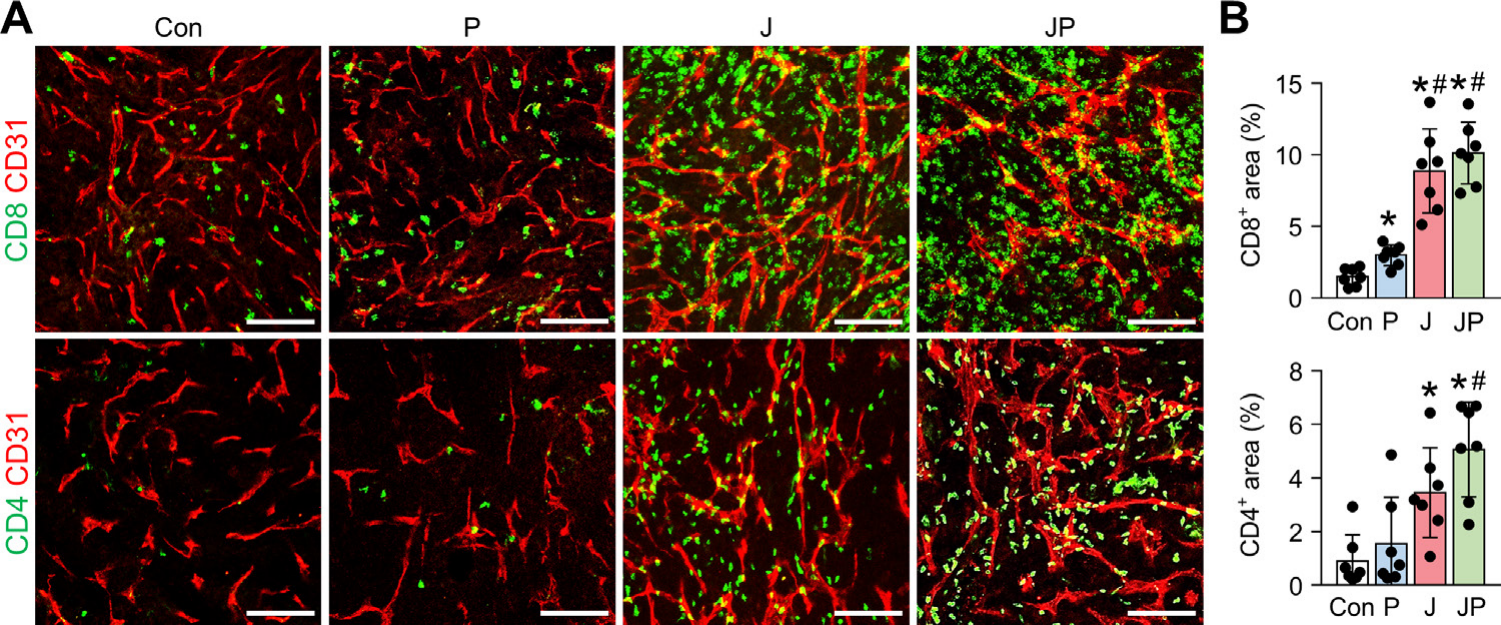
The combination immunotherapy of OV with anti-PD-1 augments the intratumoral infiltration of CD8^+^ and CD4^+^ T cells. MC38 tumor-bearing mice were intraperitoneally treated with JX and/or anti-PD-1. Representative images (A) and comparisons (B) of CD8^+^ T cells and CD4^+^ T cells in tumor. Pooled data from two experiments with n=7 per group. Values are mean±SD. *p<0.05 versus control; ^#^p<0.05 versus anti-PD-1. One-way ANOVA and Kruskal-Wallis test were used. Scale bar, 100 µm. ANOVA, analysis of variance; OV, oncolytic virotherapy; PD-1, programmed cell death protein 1.

As shown earlier, we confirmed the robust activation of peritoneal and intratumoral immunity against peritoneal metastases of colon cancer after JX treatment. However, since its therapeutic efficacy was modest as a monotherapy and could induce PD-1 upregulation in CD8^+^ T cells, we combined anti-PD-1 antibody with JX therapy to overcome the limitations. Peritoneal tumor-bearing mice were treated with either JX and/or anti-PD-1 on indicated days (figure 5A). As a result, anti-PD-1 monotherapy delayed tumor growth by 15.9%, JX monotherapy reduced tumor growth by 63.2%, and the combination therapy inhibited tumor growth by 86.3% (figure 5B, D). Mice treated with a combination immunotherapy of JX and anti-PD-1 showed a better overall survival compared with those in other groups (figure 5C). Notably, two mice in combination group experienced complete regression of peritoneal tumors and remained tumor free. Moreover, the combination therapy attenuated hemorrhage of the peritoneal tumor vessels (figure 5D, E). Besides, when the volume of malignant hemorrhagic ascites was compared, combination therapy decreased the volume of ascites by 97%, and four mice remained ascites free after the treatment (figure 5). When the TME of peritoneal tumors was analyzed, the combination therapy increased the number of tumor-infiltrating CD8^+^ T cells by 6.77-fold, whereas anti-PD-1 and JX monotherapy increased infiltrating CD8^+^ T cells by 2-fold and 5.94-fold, respectively, when compared with the controls. Moreover, the combination therapy also increased the number of tumor-infiltrating CD4^+^ T cells by 5.76-fold, whereas anti-PD-1 and JX monotherapy increased infiltrating CD4^+^ T cells by 1.71-fold and 3.37-fold, respectively when compared with the controls (figure 6A, B).

To validate the potential of combining JX and anti-PD-1 to treat PC, we examined ID8 ovarian cancer model. Mice were intraperitoneally injected with syngeneic ID8 ovarian cancer cells and treated with JX and/or anti-PD-1-antibody on indicated days (online supplemental figure S3A). When mice were sacrificed 28 days after tumor implantation, those treated with PBS showed diffuse peritoneal metastases and malignant ascites, while those treated with the combination of JX and anti-PD-1 showed suppressed peritoneal tumor dissemination by 98% and, more importantly, eradication of malignant ascites (online supplemental figure S3B–D). Overall survival was prolonged in both the JX monotherapy and JX + anti-PD-1 combination therapy groups compared with that in the control and anti-PD-1 monotherapy groups (online supplemental figure S3E). Thus, the combined immunotherapy of JX and anti-PD-1 has therapeutic potential in both ovarian cancer and colon cancer PC.

Collectively, the combination of JX with anti-PD-1 further enhanced the adaptive immune response and, therefore, resulted in the better control of peritoneal metastases and malignant ascites in advanced colon cancer.

### JX collaborates with other ICIs or antiangiogenic therapy to suppress peritoneal metastases of colon cancer

**Figure 7:**
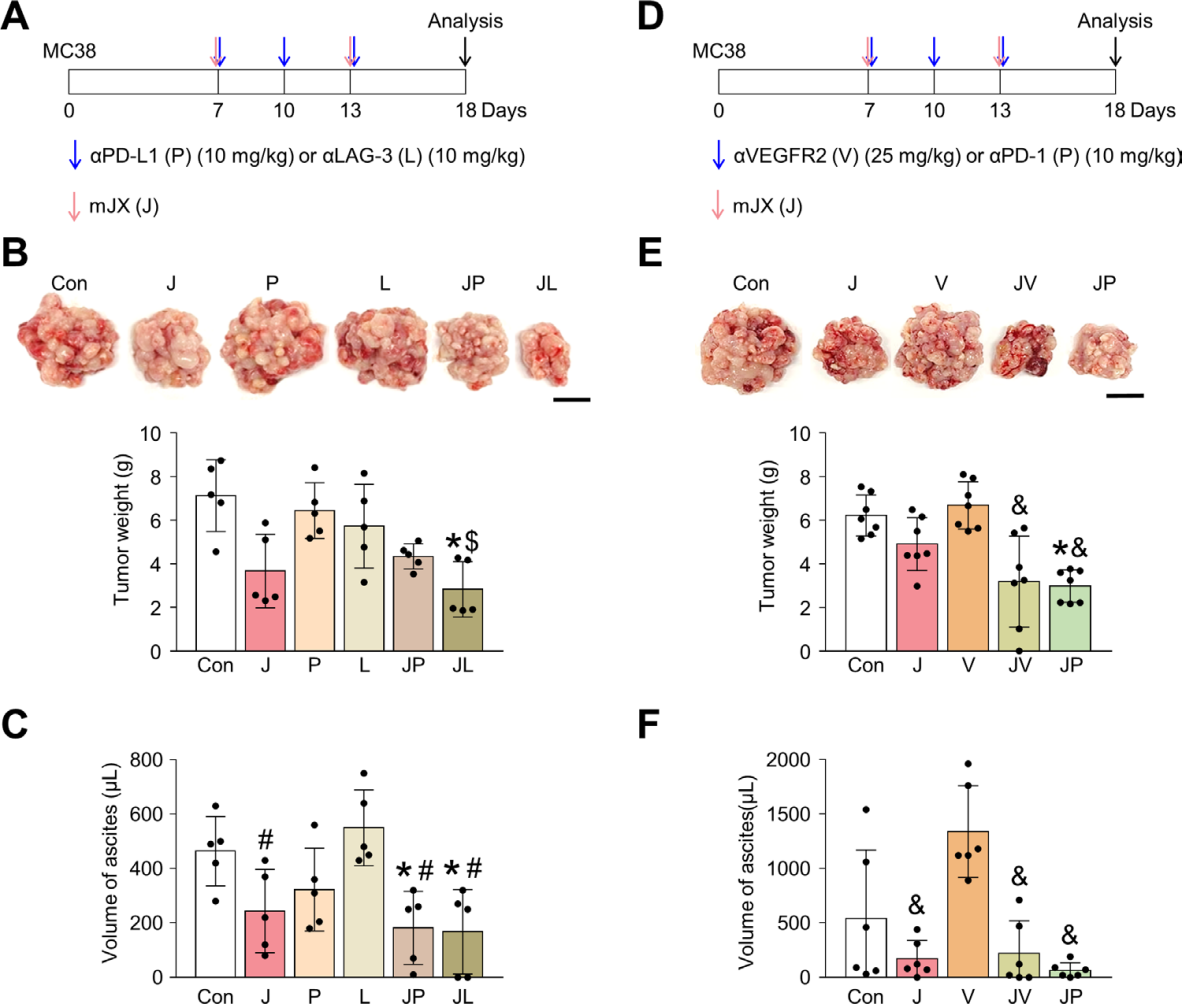
The combination treatment of JX with other ICIs or anti-VEGFR2 antibody elicits an improved antitumor effect. Mice were intraperitoneally implanted with MC38 tumor cells and treated with JX and/or ICI (anti-PD-L1, anti-LAG-3, or anti-PD-1) or anti-VEGFR2 antibody on indicated days (arrows). (A) Diagram of the treatment schedule. Red arrows indicate JX treatment. Blue arrows indicate anti-PD-L1 or anti-LAG-3 treatment. (B) Representative images and comparisons of tumor burden. (C) Representative images and comparisons of malignant ascites. (D) Diagram of the treatment schedule. Red arrows indicate JX treatment. Blue arrows indicate anti-VEGFR2 or anti-PD-1 treatment. (E) Representative images and comparisons of tumor burden. (F) Representative images and comparisons of malignant ascites. Pooled data from two experiments with n=5 to 7 per group. Values are mean±SD. *p<0.05 versus control; ^#^p<0.05 versus anti-LAG-3; ^$^p<0.05 versus anti-PD-L1; ^&^p<0.05 versus anti-VEGFR2. One-way ANOVA and Kruskal-Wallis test were used (B, C, E and F). Scale bar, 10 mm (B and E). ANOVA, analysis of variance; PD-1, programmed cell death protein 1; PD-L1, programmed death-ligand 1.

Because JX upregulated PD-L1 or LAG-3 within TME, these immune checkpoint molecules could limit the immunotherapeutic efficacy of JX monotherapy. Therefore, to further strengthen JX-based immunotherapy, we treated peritoneal MC38 tumor-bearing mice with anti-PD-L1 or anti-LAG-3 (figure 7A–C). While monotherapy with anti-PD-L1 or anti-LAG-3 showed only marginal efficacies, the combined therapy of JX to anti-PD-L1 or anti-LAG-3 revealed stronger anti-tumor efficacies and further suppressed the formation of malignant ascites within the peritoneal cavity compared with monotherapies. Therefore, the concurrent blockade of these immune checkpoints is a valid strategy to overcome the limitations of JX monotherapy.

Next, because the antiangiogenic therapy is a critical part of the current targeted therapy against advanced cancers and JX also has antiangiogenic effects, we examined the possible cooperation between these two agents for treating PC of colon cancer.^43 44^ We treated MC38 peritoneal tumor-bearing mice with anti-VEGFR2 (DC101) or anti-PD-1 (figure 7D–F). While the anti-VEGFR2 monotherapy did not show any significant efficacy in suppressing peritoneal tumor growth and malignant ascites formation, it induced stronger antitumor effects when combined with intraperitoneal JX treatment. Some mice treated with the combination of JX and anti-VEGFR2 showed complete resolution of malignant ascites within the peritoneal cavity. Moreover, the efficacy of this combination was almost comparable to that of JX and anti-PD-1 combination therapy. Thus, the combination therapy of JX and antiangiogenic agents is also effective when treating PC of colon cancer.

## Discussion

PC is a common and devastating manifestation of colon cancer which is usually accompanied by the accumulation of malignant ascites within the peritoneal cavity.^1 2 6^ In PC, a significant amount of tumor cells are free-floating within the malignant ascites or seeded widespread along the surface of the peritoneum. Since these tumor cells are poorly vascularized or not vascularized at all, the systemic delivery of conventional chemotherapeutics to peritoneal tumor cells is severely hampered, making PC as a formidable challenge when treating patients with colon cancer.^6 14^ Therefore, intraperitoneal therapy could be an attractive route of intervention, which would guarantee the direct exposure of a higher concentration of the drug for a longer duration.^6^

Here, we took advantage of intraperitoneal injection to maximize the efficacy of JX against peritoneal tumor cells. JX selectively infected and lysed peritoneal tumor cells, because it lacks thymidine kinase. It also successfully activated peritoneal DCs because it was engineered to express GM-CSF. Moreover, it reduced immunosuppressive myeloid-derived cells, restored the effector functions of CD8^+^ T cells within the peritoneal cavity and facilitated their infiltration into peritoneal tumor nodules. This JX-induced anticancer immunity effectively suppressed peritoneal tumors.

The accumulation of malignant ascites is mediated by VEGF, a pivotal proangiogenic factor, which is known to be mainly secreted from tumor cells.^12 45 46^ Excessive VEGF in the peritoneal cavity promotes robust tumor angiogenesis both on the peritoneal surface and within the peritoneal tumor nodules, generating a myriad of hyperpermeable tumor neovessels.^4 12^ In the present study, intraperitoneal JX treatment effectively suppressed the malignant ascites formation within the peritoneal cavity through multiple mechanisms. First, in our results, it destroyed peritoneal tumor cells and downregulated *Vegfa* within the tumor. Next, it promoted T cells to secrete IFN-γ, which is a potent antiangiogenic factor that impairs the proliferation and survival of endothelial cells.^39 47^ Moreover, IFN-γ could reduce the ascites formation because it is known to recruit pericytes to tumor endothelial cells, enhancing the integrity of tumor vasculatures.^47 48^

Though ICIs made a massive breakthrough in the treatment of advanced cancers, their therapeutic efficacy is limited in the treatment of PC, especially in patients with colon cancer.^19^ Consistently, in our study, ICI monotherapy revealed minimal efficacy in peritoneal tumors and malignant ascites, indicating our model is indeed an immunotherapy-resistant model. However, intraperitoneal JX treatment induced the intense infiltration of CD8^+^ T cells into the peritoneal tumors reactivated their effector functions, thus reprogramming immunotherapy-resistant peritoneal tumors into T cell-inflamed tumors that can respond well to ICI therapy. Accordingly, the combination therapy of JX and ICI (anti-PD-1, anti-PD-L1, or anti-LAG-3) showed dramatic control of peritoneal metastases and malignant ascites, even leading to complete regression in some mice.

Intriguingly, the JX-based combination immunotherapy remarkably repressed malformed vasculatures on the surface of the peritoneum. Recently, the mutual regulation of tumor vasculatures and anticancer immunity is increasingly recognized as an essential determinant of tumor progression.^20 48–50^ Tumor angiogenesis disturbs anticancer immunity, whereas anticancer immunity suppresses tumor angiogenesis.^48^ Therefore, a robust anticancer immunity elicited by combination immunotherapy would contribute to the suppressed tumor angiogenesis in the peritoneum.

## Conclusions

In conclusion, our study demonstrated that intraperitoneal immunotherapy with JX activates peritoneal anticancer immunity and potentiate immune checkpoint blockade to suppress PC and malignant ascites in colon cancer.

## References

[1] Ceelen W, Ramsay RG, Narasimhan V, et al Targeting the tumor microenvironment in colorectal peritoneal metastases. Trends Cancer 2020;6:236–46. 10.1016/j.trecan.2019.12.008 32101726

[2] Lemoine L, Sugarbaker P, Van der Speeten K Pathophysiology of colorectal peritoneal carcinomatosis: role of the peritoneum. World J Gastroenterol 2016;22:7692–707. 10.3748/wjg.v22.i34.7692 27678351 PMC5016368

[3] Sedlacek AL, Gerber SA, Randall TD, et al Generation of a dual-functioning antitumor immune response in the peritoneal cavity. Am J Pathol 2013;183:1318–28. 10.1016/j.ajpath.2013.06.030 23933065 PMC3791689

[4] Melichar B, Freedman RS Immunology of the peritoneal cavity: relevance for host-tumor relation. Int J Gynecol Cancer 2002;12:3–17. 10.1046/j.1525-1438.2002.01093.x 11860531

[5] Yoneda A et al Immunological milieu in the peritoneal cavity at laparotomy for gastric cancer. World J Gastroenterol 2012;18:1470–8. 10.3748/wjg.v18.i13.1470 22509078 PMC3319942

[6] Ströhlein MA, Heiss MM, Jauch K-W The current status of immunotherapy in peritoneal carcinomatosis. Expert Rev Anticancer Ther 2016;16:1019–27. 10.1080/14737140.2016.1224666 27530056

[7] Mikuła-Pietrasik J, Uruski P, Tykarski A, et al The peritoneal “soil” for a cancerous “seed”: a comprehensive review of the pathogenesis of intraperitoneal cancer metastases. Cell. Mol. Life Sci. 2018;75:509–25. 10.1007/s00018-017-2663-1 28956065 PMC5765197

[8] Klaver YLB, Lemmens VE, Nienhuijs SW Peritoneal carcinomatosis of colorectal origin: incidence, prognosis and treatment options. World J Gastroenterol 2012;18:5489–94. 10.3748/wjg.v18.i39.5489 23112540 PMC3482634

[9] Abiko K, Mandai M, Hamanishi J, et al PD-L1 on tumor cells is induced in ascites and promotes peritoneal dissemination of ovarian cancer through CTL dysfunction. Clin Cancer Res 2013;19:1363–74. 10.1158/1078-0432.CCR-12-2199 23340297

[10] Tseng S-H, Park S-T, Lam B, et al Novel, genetically induced mouse model that recapitulates the histological morphology and immunosuppressive tumor microenvironment of metastatic peritoneal carcinomatosis. J Immunother Cancer 2020;8:e000480 10.1136/jitc-2019-000480 32111730 PMC7057437

[11] Olszewski WL, Kubicka U, Tarnowski W, et al Activation of human peritoneal immune cells in early stages of gastric and colon cancer. Surgery 2007;141:212–21. 10.1016/j.surg.2006.06.031 17263978

[12] Kobold S, Hegewisch‐Becker S, Oechsle K, et al Intraperitoneal VEGF inhibition using bevacizumab: a potential approach for the symptomatic treatment of malignant ascites? Oncologist 2009;14:1242–51. 10.1634/theoncologist.2009-0109 20008305

[13] Pogge von Strandmann E, Reinartz S, Wager U, et al Tumor-Host cell interactions in ovarian cancer: pathways to therapy failure. Trends Cancer 2017;3:137–48. 10.1016/j.trecan.2016.12.005 28718444

[14] Aoyagi T, Terracina KP, Raza A Current treatment options for colon cancer peritoneal carcinomatosis. World J Gastroenterol 2014;20:12493–500. 10.3748/wjg.v20.i35.12493 25253949 PMC4168082

[15] Ceelen W HIPEC with oxaliplatin for colorectal peritoneal metastasis: the end of the road? Eur J Surg Oncol 2019;45:400–2. 10.1016/j.ejso.2018.10.542 30392745

[16] Narasimhan V et al Colorectal peritoneal metastases: pathogenesis, diagnosis and treatment options - an evidence-based update. ANZ J Surg 2020. 10.1111/ans.15796 32129577

[17] Lau J, Cheung J, Navarro A, et al Tumour and host cell PD-L1 is required to mediate suppression of anti-tumour immunity in mice. Nat Commun 2017;8:14572 10.1038/ncomms14572 28220772 PMC5321797

[18] Topalian SL, Drake CG, Pardoll DM Immune checkpoint blockade: a common denominator approach to cancer therapy. Cancer Cell 2015;27:450–61. 10.1016/j.ccell.2015.03.001 25858804 PMC4400238

[19] Chon HJ, Kim H, Noh JH, et al STING signaling is a potential immunotherapeutic target in colorectal cancer. J Cancer 2019;10:4932–8. 10.7150/jca.32806 31598165 PMC6775531

[20] Lee WS, Yang H, Chon HJ, et al Combination of anti-angiogenic therapy and immune checkpoint blockade normalizes vascular-immune crosstalk to potentiate cancer immunity. Exp Mol Med 2020;52:1475–85. 10.1038/s12276-020-00500-y 32913278 PMC8080646

[21] Ribas A, Wolchok JD Cancer immunotherapy using checkpoint blockade. Science 2018;359:1350–5. 10.1126/science.aar4060 29567705 PMC7391259

[22] Engeland CE, Grossardt C, Veinalde R, et al CTLA-4 and PD-L1 checkpoint blockade enhances oncolytic measles virus therapy. Mol Ther 2014;22:1949–59. 10.1038/mt.2014.160 25156126 PMC4429737

[23] Liu Z, Ravindranathan R, Kalinski P, et al Rational combination of oncolytic vaccinia virus and PD-L1 blockade works synergistically to enhance therapeutic efficacy. Nat Commun 2017;8:14754 10.1038/ncomms14754 28345650 PMC5378974

[24] Kim CG, Kim C, Yoon SE, et al Hyperprogressive disease during PD-1 blockade in patients with advanced hepatocellular carcinoma. J Hepatol 2020. 10.1016/j.jhep.2020.08.010. [Epub ahead of print: 15 Aug 2020]. 32810553

[25] Ma Z, Li W, Yoshiya S, et al Augmentation of immune checkpoint cancer immunotherapy with IL18. Clin Cancer Res 2016;22:2969–80. 10.1158/1078-0432.CCR-15-1655 26755531

[26] Russell SJ, Peng K-W, Bell JC Oncolytic virotherapy. Nat Biotechnol 2012;30:658–70. 10.1038/nbt.2287 22781695 PMC3888062

[27] Kaufman HL, Kohlhapp FJ, Zloza A Oncolytic viruses: a new class of immunotherapy drugs. Nat Rev Drug Discov 2015;14:642–62. 10.1038/nrd4663 26323545 PMC7097180

[28] Bartlett DL, Liu Z, Sathaiah M, et al Oncolytic viruses as therapeutic cancer vaccines. Mol Cancer 2013;12:103 10.1186/1476-4598-12-103 24020520 PMC3847443

[29] Breitbach CJ, Lichty BD, Bell JC Oncolytic viruses: therapeutics with an identity crisis. EBioMedicine 2016;9:31–6. 10.1016/j.ebiom.2016.06.046 27407036 PMC4972563

[30] Koske I, Rössler A, Pipperger L, et al Oncolytic virotherapy enhances the efficacy of a cancer vaccine by modulating the tumor microenvironment. Int. J. Cancer 2019;145:1958–69. 10.1002/ijc.32325 30972741 PMC6767478

[31] Chan WM, McFadden G Oncolytic poxviruses. Annu Rev Virol 2014;1:191–214. 10.1146/annurev-virology-031413-085442 PMC4380149 25839047

[32] Thorne SH Immunotherapeutic potential of oncolytic vaccinia virus. Front Oncol 2014;4:155 10.3389/fonc.2014.00155 24987615 PMC4060052

[33] Heo J, Reid T, Ruo L, et al Randomized dose-finding clinical trial of oncolytic immunotherapeutic vaccinia JX-594 in liver cancer. Nat Med 2013;19:329–36. 10.1038/nm.3089 23396206 PMC4268543

[34] Breitbach C, Bell JC, Hwang T-H, et al The emerging therapeutic potential of the oncolytic immunotherapeutic Pexa-Vec (JX-594). Oncolytic Virother 2015;4:25–31. 10.2147/OV.S59640 27512667 PMC4918374

[35] Hernandez-Gea V, Alsinet C, Llovet JM Oncolytic immunotherapeutic virus in HCC: can it compete with molecular therapies? J Hepatol 2013;59:882–4. 10.1016/j.jhep.2013.05.006 23673136

[36] Chon HJ, Lee WS, Yang H, et al Tumor microenvironment remodeling by intratumoral oncolytic vaccinia virus enhances the efficacy of Immune-Checkpoint blockade. Clin Cancer Res 2019;25:1612–23. 10.1158/1078-0432.CCR-18-1932 30538109

[37] Breitbach CJ, Arulanandam R, De Silva N, et al Oncolytic vaccinia virus disrupts tumor-associated vasculature in humans. Cancer Res 2013;73:1265–75. 10.1158/0008-5472.CAN-12-2687 23393196

[38] Lun X, Chan J, Zhou H, et al Efficacy and safety/toxicity study of recombinant vaccinia virus JX-594 in two immunocompetent animal models of glioma. Mol Ther 2010;18:1927–36. 10.1038/mt.2010.183 20808290 PMC2990519

[39] Yang H, Lee WS, Kong SJ, et al STING activation reprograms tumor vasculatures and synergizes with VEGFR2 blockade. J Clin Invest 2019;129:4350–64. 10.1172/JCI125413 31343989 PMC6763266

[40] Breitbach CJ, De Silva NS, Falls TJ, et al Targeting tumor vasculature with an oncolytic virus. Mol Ther 2011;19:886–94. 10.1038/mt.2011.26 21364541 PMC3098639

[41] Fucikova J, Becht E, Iribarren K, et al Calreticulin expression in human non-small cell lung cancers correlates with increased accumulation of antitumor immune cells and favorable prognosis. Cancer Res 2016;76:1746–56. 10.1158/0008-5472.CAN-15-1142 26842877

[42] Liu X, Li J, Liu Y, et al Calreticulin acts as an adjuvant to promote dendritic cell maturation and enhances antigen-specific cytotoxic T lymphocyte responses against non-small cell lung cancer cells. Cell Immunol 2016;300:46–53. 10.1016/j.cellimm.2015.12.003 26702740

[43] Kanat O, Ertas H Existing anti-angiogenic therapeutic strategies for patients with metastatic colorectal cancer progressing following first-line bevacizumab-based therapy. World J Clin Oncol 2019;10:52–61. 10.5306/wjco.v10.i2.52 30815371 PMC6390122

[44] Kim C, Yang H, Fukushima Y, et al Vascular RhoJ is an effective and selective target for tumor angiogenesis and vascular disruption. Cancer Cell 2014;25:102–17. 10.1016/j.ccr.2013.12.010 24434213

[45] Lee J-E, Kim C, Yang H, et al Novel glycosylated VEGF decoy receptor fusion protein, VEGF-Grab, efficiently suppresses tumor angiogenesis and progression. Mol Cancer Ther 2015;14:470–9. 10.1158/1535-7163.MCT-14-0968-T 25534360

[46] Park J-S, Kim I-K, Han S, et al Normalization of tumor vessels by Tie2 activation and Ang2 inhibition enhances drug delivery and produces a favorable tumor microenvironment. Cancer Cell 2016;30:953–67. 10.1016/j.ccell.2016.10.018 27960088

[47] De Palma M, Biziato D, Petrova TV Microenvironmental regulation of tumour angiogenesis. Nat Rev Cancer 2017;17:457–74. 10.1038/nrc.2017.51 28706266

[48] Tian L, Goldstein A, Wang H, et al Mutual regulation of tumour vessel normalization and immunostimulatory reprogramming. Nature 2017;544:250–4. 10.1038/nature21724 28371798 PMC5788037

[49] Haro MA, Dyevoich AM, Phipps JP, et al Activation of B-1 cells promotes tumor cell killing in the peritoneal cavity. Cancer Res 2019;79:159–70. 10.1158/0008-5472.CAN-18-0981 30224373 PMC6318009

[50] De Palma M, Jain RK CD4+ T cell activation and vascular normalization: two sides of the same coin? Immunity 2017;46:773–5. 10.1016/j.immuni.2017.04.015 28514684

