# Supplementary material for: Oncolytic vaccinia virus reinvigorates peritoneal immunity and cooperates with immune checkpoint inhibitor to suppress peritoneal carcinomatosis in colon cancer
Source: J Immunother Cancer. 2020 Nov 16;8(2):e000857. doi: 10.1136/jitc-2020-000857 (PMC7670945; doi:10.1136/jitc-2020-000857)
Supplement: Supplementary data [file jitc-2020-000857supp001.pdf]

Supplementary data

Oncolytic vaccinia virus reinvigorates peritoneal immunity and cooperates with immune checkpoint inhibitor to suppress peritoneal carcinomatosis in colon cancer

Yu Seong Lee, Won Suk Lee, Chang Woo Kim, Seung Joon Lee, Hannah Yang, So Jung Kong, John Ning, Kyung-Mee Yang, Beodeul Kang, Woo Ram Kim, Hong Jae Chon*, Chan Kim*

* Corresponding author. e-mail: minidoctor@cha.ac.kr and chan@cha.ac.kr

Supplementary Figures

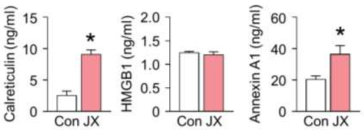

Supplementary Figure 1. JX-infected tumor cells produce damage-associated molecular patterns.

Comparisons of calreticulin, HMGB1, and annexin A1 levels in PBS- and JX-treated MC38 tumor cell supernatants. Calreticulin and annexin A1 (left and right panel) was markedly upregulated, whereas HMGB1 (middle panel) was not difference compare to control.

Pooled data from two experiments with n = 7 per group. Values are mean ± SD. *P < 0.05 versus control. Two-tailed Student's t-test was used.

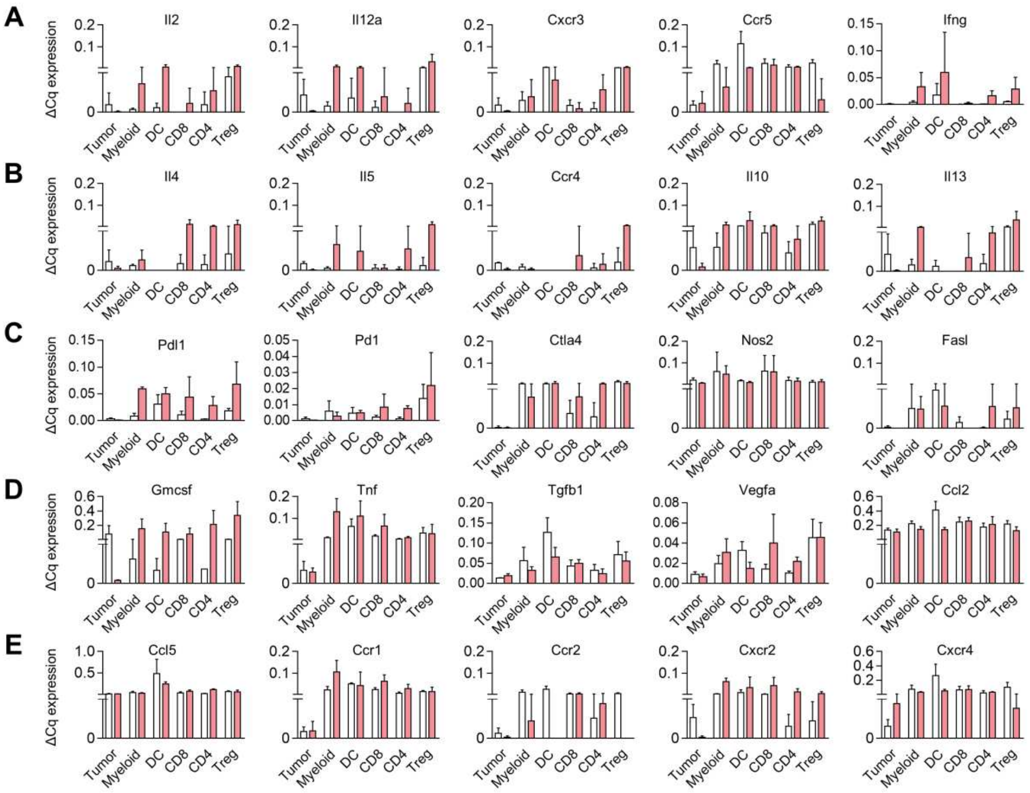

Supplementary Figure 2. JX regulates tumor microenvironment by expressing immune-related genes in different cell types.

MC38 tumor-bearing mice were intraperitoneally treated with PBS or JX. Tumor cells ($CD45^-CD31^-$), $CD4^+$ T cell ($CD45^+CD4^+$), $CD8^+$ T cell ($CD45^+CD8^+$), DCs ($CD45^+CD11c^+$), myeloid cell ($CD45^+CD11b^+$) and Tregs ($CD4^+CD25^+$) were sorted from tumors using MoFlo XDP cell sorter.

(A-E) The quantitative analysis of immune-related genes in FACS-sorted tumor cells, myeloid cells, dendritic cells (DCs), $CD8^+$ T cells, $CD4^+$ T cells, and regulatory T cells (Treg) from peritoneal tumors. Pooled data from two experiments with n = 3 per group.

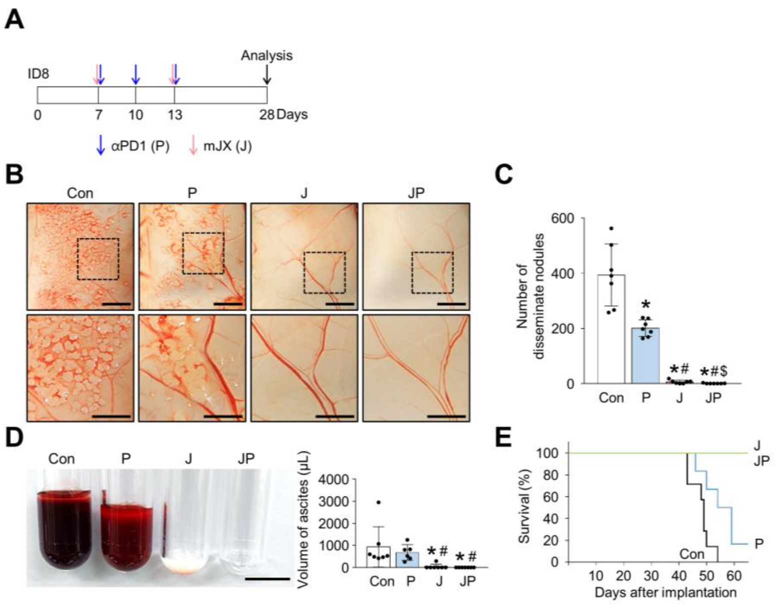

Supplementary Figure 3. The combination immunotherapy of JX with anti-PD-1 inhibits peritoneal carcinomatosis and malignant ascites formation in ID8 ovarian cancer.

Mice were intraperitoneally implanted with ID8 tumor cells and treated with JX and/or anti-PD-1 on indicated days (arrows). Red arrows indicate JX treatment. Blue arrows indicate anti-PD-1 treatment.

(A) Diagram of the treatment schedule.

(B) Representative images and comparisons of disseminated tumor nodules.

(C) Comparison of the number of disseminated tumor nodules.

(D) Representative images and comparisons of malignant ascites.

(E) Kaplan–Meier survival curves for overall survival.

Pooled data from two experiments with n = 6 to 7 per group. Values are mean ± SD. $^{*}P < 0.05$ versus control; $^{\#}P < 0.05$ versus αPD-1; $^{\$}P < 0.05$ versus JX. Two-tailed Student’s *t*-test was used (C and D). Scale bar, 5 mm (B) and 10 mm (D).
